# Circulating Metabolomic Signature in Generalized Pustular Psoriasis Blunts Monocyte Hyperinflammation by Triggering Amino Acid Response

**DOI:** 10.3389/fimmu.2021.739514

**Published:** 2021-09-08

**Authors:** Ning Yu, Chen Peng, Wenjuan Chen, Ziwen Sun, Jianfeng Zheng, Shujie Zhang, Yangfeng Ding, Yuling Shi

**Affiliations:** ^1^Department of Dermatology, Shanghai Skin Disease Hospital, Institute of Psoriasis, Tongji University, Shanghai, China; ^2^Eye Institute and Department of Ophthalmology, Eye & ENT Hospital, Fudan University, NHC Key Laboratory of Myopia, Chinese Academy of Medical Sciences, Shanghai Key Laboratory of Visual Impairment and Restoration, Shanghai, China

**Keywords:** generalized pustular psoriasis, metabolomics, amino acid, monocyte, GCN2 2, IL-1β

## Abstract

Generalized pustular psoriasis (GPP), the most grievous variant of psoriasis, is featured by dysregulated systemic inflammatory response. The cellular and molecular basis of GPP is poorly understood. Blood monocytes are key players of host defense and producers of inflammatory cytokines including IL-1β. How the immune response of monocytes is affected by metabolic internal environment in GPP remains unclear. Here, we performed a metabolomic and functional investigation of GPP serum and monocytes. We demonstrated a significant increase in IL-1β production from GPP monocytes. In GPP circulation, serum amyloid A (SAA), an acute-phase reactant, was dramatically increased, which induced the release of IL-1β from monocytes in a NLRP3-dependent manner. Using metabolomic analysis, we showed that GPP serum exhibited an amino acid starvation signature, with glycine, histidine, asparagine, methionine, threonine, lysine, valine, isoleucine, tryptophan, tyrosine, alanine, proline, taurine and cystathionine being markedly downregulated. In functional assay, under amino acid starvation condition, SAA-stimulated mature IL-1β secretion was suppressed. Mechanistically, at post-transcriptional level, amino acid starvation inhibited the SAA-mediated reactive oxygen species (ROS) formation and NLRP3 inflammasome activation. Moreover, the immune-modulatory effect of amino acid starvation was blocked by silencing general control nonderepressible 2 kinase (GCN2), suggesting the involvement of amino acid response (AAR) pathway. Collectively, our results suggested that decreased serum amino acids in GPP blunted the innate immune response in blood monocytes through AAR pathway, serving as a feedback mechanism preventing excessive inflammation in GPP.

## Introduction

Generalized pustular psoriasis (GPP) is an uncommon and life-threatening disease, which is featured by generalized erythema, desquamation, and dramatic cutaneous neutrophilic infiltration (1). There is a significant systemic inflammatory component in GPP including fever, fatigue and neutrophilia. Up to now, the immunopathogenesis of GPP remains unclear.

IL-1 is one of the dominant cytokines in GPP autoinflammation (2, 3). The pathological role of IL-1β in psoriasis has been reported (4, 5). Recently, it was reported that GPP patients treated with anti-IL-1β monoclonal antibodies had dramatic clinical improvement (6, 7). As one of major producers of IL-1β, monocytes initiate and promote inflammation in the context of sterile and sterile injury, and during autoinflammatory diseases (8, 9). During inflammation, secretion of IL-1β must be tightly controlled to avoid tissue damage. However, it would be of critical importance to investigate the production and regulation mechanism of IL-1β in GPP.

The mature form of IL-1β is regulated transcriptionally and post-transcriptionally (10, 11).

NF-κB mediates the transcription of pro-IL-1β gene, while inflammasome promotes the production of IL-1β at post-transcriptional level. In the process of infection and tissue injury, serum amyloid A (SAA), an acute-phase reactant, is highly increased in circulation (12–14). Previously, we reported that epidermis-derived SAA was a trigger of IL-1β secretion from keratinocytes (15). However, the impact of SAA on circulatory immune cells is largely unknown.

Metabolomics refers to the identification and analysis of multiple end products in metabolism. The systemic metabolism is closely related to the pathological process of disease (16, 17). In previous metabolomic researches, metabolite perturbations in psoriasis vulgaris (PV) have been identified. The metabolite profiles might serve as potential biomarkers of PV severity and predictors of therapeutic response (18–24). However, the metabolomic signature of GPP and its effects on hyperinflammation in GPP remains to be investigated.

Here, we have applied serum non-targeted and targeted metabolomic analysis in patients with GPP. A significant decrease of overall levels of serum amino acids were observed in GPP patients. In functional study, we demonstrated that amino acid starvation dampened NACHT, LRR and PYD domains-containing protein 3 (NLRP3) inflammasome activation and IL-1β production induced by SAA in monocytes. The subsequent mechanistic study revealed a critical role for general control nonderepressible-2 kinase (GCN2) activation underlying the immune-modulatory effect of amino acid starvation. Our data suggested that amino acid starvation might function as a feedback mechanism preventing the hyperinflammation in GPP progression.

## Materials and Methods

### Subjects

24 GPP patients, 12 PV patients and 12 healthy controls (HC) were recruited between April 2017 and October 2019. All the patients were Chinese and inpatients at Shanghai Skin Disease Hospital of Tongji University. Clinical information on age, gender, body mass index (BMI), serum albumin, C-reactive protein (CRP), serum calcium, lymphocyte count, neutrophil count, neutrophil/lymphocyte ratio, arthritis and nonalcoholic fatty liver disease (NAFLD) status were recorded. The GPP severity score in each case was calculated with reference to the Japanese Dermatological Association (JDA) severity score for GPP. Serum samples from patients and HC were collected and stored at -80°C.

### Non-Targeted Metabolomic Profiling and Data Extraction

Metabolite extraction and gas chromatography-mass spectrometry (GC-MS) were carried out as described previously (25). 200 μl of methanol was mixed with each sample to precipitate proteins and then left standing at room temperature for 10 min. 150 μl supernatant was obtained by centrifugation and then dried in the nitrogen stream at 40°C. The dried samples were mixed with methoxyamine pyridine solution. We performed an oximation reaction and silylation reaction in 40°C oven, then we centrifuged the solution following the derivatization reaction. Finally, we analyzed the supernatant using GC-MS.

We then analyzed the samples with GC-MS based on an Agilent 7890B GC system linked to an Agilent 5977A MSD system (Agilent Technologies Inc, Folsom, California). The carrier gas was Helium (1 ml/ml). The injector temperature and volume were 260°C and 1 µl. To get a better sensitivity of the chromatographic process, the adopted temperature procedure was: the initial 60°C, to 125°C (8°C/min), to 210°C (5°C/min), to 270°C (10°C/min), to 305°C (20°C/min) and 305°C (5 min). In addition, the temperature of MS quadrupole was 150°C, while ion source was 230°C. We carried out the detection using electron impact ionization (70 eV) in the full-scan monitor mode (m/z 50-500). Then we randomized the injection sequence and used a quality control (QC) to verify the quality of the data. We obtained the crude data sets comprising peak names, sample information, peak area, retention time, and quality score ratio. After deleting all the pseudo positive peaks, the data were normalized. The peaks of the same compounds were combined.

### Metabolomic Data Analysis

The differential metabolite identification was performed using the Progenesis QI software (Waters Corporation, Milford, USA) and the metabolome database of Shanghai Lu-Ming Biotech Co. Ltd (Shanghai, China). Univariate assay was adopted to determine significantly perturbed metabolites between GPP patients and healthy controls using two-sample t-test. Crude *P* was adjusted by Benjamini and Hochberg method. We performed a false discovery rate (FDR) control in multiple comparisons. For multivariate assay, we carried out principal component analysis (PCA) and orthogonal partial least squares discriminant analysis (OPLS-DA). R^2^ and Q^2^ scores were obtained to examine the model validity, which was further verified by the permutation test. In OPLS-DA model, variable importance in the projection (VIP) values were calculated, while *P*-values were examined in t-test. In the comparison between GPP and HC, those metabolites with VIP > 1.0 and P-values < 0.05 were deemed to be significantly different.

### Weighted Correlation Network Analysis (WGCNA)

Network and clustering analysis were performed using the WGCNA R package (26). Topological overlap matrix (TOM) was generated from adjacency matrix. Based on the TOM‐based dissimilarity, we allocated metabolites into 3 modules. In order to determine critical modules, minimal module size was set as 20, cut height as 0.25, and soft-thresholding power as 3 (scale free R^2^ = 0.9). The module with the strongest association with disease status was chosen to investigate the biological function by pathway enrichment analysis. Associations among metabolites within the blue module were projected as nodes and edges with Cytoscape (version 3.8).

### Pathway Enrichment Analysis

Pathway enrichment analysis was performed on metabolites within the blue module in WGCNA for further biological interpretation. For pathway topology assay, we calculated the relative-betweenness centrality. Pathway enrichment analysis (*P*-values < 0.05) and pathway topology analysis (impact values > 0.1) were used to choose potential targets. Identification of pathways was carried out utilizing MetaboAnalyst 4.0 (27).

### Amino Acid-Targeted Metabolomics

The levels of serum amino acids were measured using ultra-performance liquid chromatography-tandem-mass spectrometry (UPLC-MS/MS) as reported previously (28). UPLC separation was carried out by an Agilent 1290 Infinity II series UPLC System (Agilent Technologies), equipped with a Waters ACQUITY UPLC BEH Amide column. A gradient elution program was used in the UPLC system. The metabolite analysis was performed using Agilent 6460 triple quadrupole mass spectrometer (Agilent Technologies). For quantification, the optimal multiple reaction monitoring (MRM) parameters for each metabolite were determined. Agilent MassHunter Work Station Software (Agilent Technologies) was adopted to acquire and process the MRM data.

### Monocyte Isolation and Culture Conditions

We isolated human monocytes using Ficoll-Paque density-gradient centrifugation and EasySep Direct Human Monocyte Isolation Kit (Stemcell Technologies, Grenoble, France). The monocytes were cultured in the presence or absence of SAA (0.01-10 μg/ml; Peprotech, Rocky Hills, NJ, USA) in either amino acid-free (AAF) medium or normal DMEM medium for up to 24 h. The “AAF” culture indicate amino acid-free DMEM (USBiological, Massachusetts) plus 10% FBS.

### Enzyme-Linked Immunosorbent Assay (ELISA)

Monocytes were seeded in 12-well plates. After treatment, we collected the culture supernatants and pelleted cell debris by centrifuging. IL-1β ELISA kit (R&D systems, Minneapolis, MN, USA) was used to analyze the supernatants. Serum SAA levels were evaluated by ELISA kit (R&D systems).

### Real-Time RT-PCR

RNA isolation and real-time RT-PCR was performed as described earlier (15). Sample data are presented as fold induction compared to control cells.

### Flow Cytometry

The monocytes were pretreated with normal medium or AAF medium overnight, and then stimulated with 10 μg/ml SAA for 15 min. Phosphorylation of NF-κB 65 was analyzed by flow cytometry as described previously (15). In short, the monocytes were fixed and permeabilized.

We then stained the monocytes with phycoerythrin (PE)-labelled phospho-p65 antibody (BD Biosciences). LSRII FACS analyzer (BD Biosciences) was used to analyze the stained monocytes. To measure ROS levels, treated monocytes were cultured with CM-H2DCFDA (Invitrogen, Carlsbad, CA, USA) for 30 min at 37°C, and tested by flow cytometry.

### Western Blot

We collected treated monocytes and lysed them with RIPA buffer. Electrophoresis with 8%-12% SDS-polyacrylamide gel was used to separate proteins. We then transferred the proteins onto a PVDF membrane (Millipore, Bedford, MA). After blocking the membrane with 2% BSA for 1 hour at room temperature, we incubated the membrane overnight at 4°C with the primary antibodies against NLRP3 (ab263899, Abcam), cleaved caspase-1 (D57A2, Cell Signaling Technology), GCN2 (ab134053, Abcam), phospho-GCN2 (ab75836, Abcam) and β-actin (ab8227, Abcam). We probed the membranes with horseradish peroxidase-conjugated secondary antibodies, and then developed the membranes with an enhanced chemiluminescence system.

### Knockdown of NLRP3 and GCN2 Expression

To knockdown endogenous NLRP3 and GCN2 expression, transfection of siRNA oligonucleotides (Santa Cruz Biotechnology, Santa Cruz, CA, USA) were adopted as described previously (15). The healthy monocytes were transfected with either a siRNA targeting NLRP3, GCN2 or a control siRNA and cultured for 7 h at 37°C. The monocytes were then stimulated with SAA (10 μg/ml) in the presence of AAF medium for 24 h, the supernatant was collected for IL-1β ELISA analysis.

### Statistical Analysis

All statistical analyses were performed using GraphPad Prism 9 or the R (http://www.R-project.org). Unpaired Student’s t-test or one-way ANOVA with Dunnett’s *post hoc* test were used to determine the significance of differences between groups. All data represent the mean ± SD. *P* < 0.05 was considered a statistically significant difference.

## Results

### Circulating SAA Induces IL-1β Secretion From GPP Monocytes

**Figure 1:**
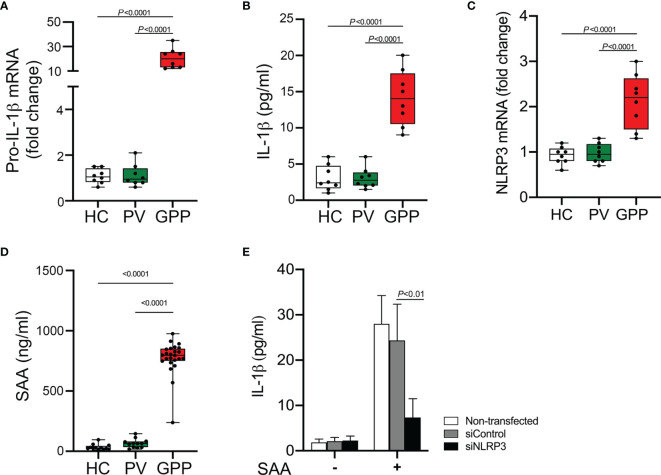
Production of IL-1β from blood monocytes induced by SAA. **(A)** Blood monocytes were isolated from GPP, PV and HC. Pro-IL-1β mRNA levels were measured by real-time RT-PCR. **(B)** Monocytes from GPP (n = 8), PV (n = 8) and HC (n = 8) were cultured *ex vivo* for 24 h, and then IL-1β in the supernatants was quantified by ELISA. **(C)** NLRP3 mRNA fold change in monocytes from GPP (n = 8), PV (n = 8) and HC (n = 8) were examined using real-time RT-PCR. **(D)** Serum levels of SAA in patients with GPP (n = 24), PV (n = 12), and HC (n = 12). **(E)** Healthy monocytes were transfected with siRNA targeting NLRP3 (siNLRP3) or a non-specific siRNA (siControl), and then cultured with SAA (10 μg/ml) for 24 h The supernatants were tested with IL-1β ELISA. Data are presented as mean ± SD, and are assessed by One-way ANOVA and Dunnett’s *post hoc* test.

Monocytes are producers of a variety of inflammatory cytokines, thus a major contributor in cytokine storm (29). However, whether monocytes can actually contribute to the pathogenesis of GPP needs investigation. Therefore, we sought to examine the mRNA level of IL-1β (pro-IL-1β mRNA) in human blood monocytes isolated from GPP, PV patients and HC. Pro-IL-1β mRNA was upregulated in GPP monocytes compared to that in PV and HC monocytes (**Figure 1A**). Analysis of monocyte culture supernatants confirmed the significant upregulation of IL-1β in GPP group, as compared to PV and HC groups (**Figure 1B**). Moreover, NLRP3 mRNA was also upregulated in GPP monocytes, suggesting NLRP3 inflammasome activation (**Figure 1C**).

Previous reports and our work suggested that SAA was an inducer of IL-1β production (15, 30). Here, we tested serum SAA levels in GPP and PV as well as HC. The levels of serum SAA in GPP patients were significantly higher than PV patients and HC, while the difference between PV and HC groups was not significant (**Figure 1D**).

We postulated that elevated SAA in GPP serum might stimulate IL-1β production in monocytes. To explore this speculation, healthy monocytes were cultured with SAA, and then IL-1β secretion was measured. As shown in **Figure 1E**, SAA significantly enhanced the expression of IL-1β. Moreover, NLRP3 knockdown by siRNA significantly suppressed SAA-mediated IL-1β release, suggesting an involvement of NLRP3 inflammasome in the process.

### GPP Serum Exhibits a Distinct Metabolomic Signature

During cytokine storm in GPP, the immune function of monocytes is affected and regulated by the mediators in circulation. We speculated that the end-point products of metabolism in circulation might be possible candidates. Therefore, we explored the metabolomic signature in GPP serum. 24 GPP patients, 12 PV patients and 12 HC were recruited. The prevalence of general factors such as age, gender, BMI, as well as comorbidities like arthritis and NAFLD were not significantly different among the three groups (**Table 1**). The serum albumin level, CRP, neutrophil count, neutrophil/lymphocyte ratio in GPP were higher than healthy controls, while serum calcium and lymphocyte count were lower in GPP patients.

**Table 1:** Baseline demographics and disease characteristics.

	GPP patients (n = 24)	PV patients (n = 12)	HCs (n = 12)
Age (years), mean ± SD	46.2 ± 14.1	42.0 ± 14.4	40.6 ± 13.2
Male, % (n)	25.0 (6)	25.0 (3)	33.3 (4)
BMI (Kg/m^2^), mean ± SD	24.5 ± 6.7	24.2 ± 6.7	23.4 ± 4.8
Albumin (g/L), mean ± SD	34.0 ± 5.5^***^	47.0 ± 2.5	45.9 ± 3.3
CRP (mg/L), mean ± SD	89.0 ± 55.1^***^	3.2 ± 1.4	3.3 ± 1.2
Calcium (mmol/L), mean ± SD	2.1 ± 0.2^***^	2.4 ± 0.1	2.5 ± 0.2
Lymphocyte count (×10^9^/L), mean ± SD	1.3 ± 0.5^*^	1.9 ± 0.7	1.9 ± 0.3
Neutrophil count (×10^9^/L), mean ± SD	9.3 ± 4.1^***^	4.8 ± 2.1	4.4 ± 2.4
Neutrophil/Lymphocyte, mean ± SD	9.0 ± 7.4^***^	2.7 ± 1.0	2.5 ± 1.4
PASI, mean ± SD	/	21.4 ± 5.6	/
Severity index, median (range)	11.5 (8-16)	/	/
Psoriatic arthritis, % (n)	45.8 (11)	25 (3)	/
NAFLD, % (n)	41.7 (10)	33.3 (4)	41.7 (5)

GPP, generalized pustular psoriasis; PV, psoriasis vulgaris; HCs, healthy controls; BMI, body mass index; CRP, C-reactive protein; PASI, psoriasis area and severity index; NAFLD, nonalcoholic fatty liver disease; ^*^P < 0.05 and ^***^P < 0.001 for the difference between GPP and HC, as well as difference between PV patients and healthy controls. P-values are calculated according to ANOVA (continuous data) or Fisher’s exact test (categorical data).

**Figure 2:**
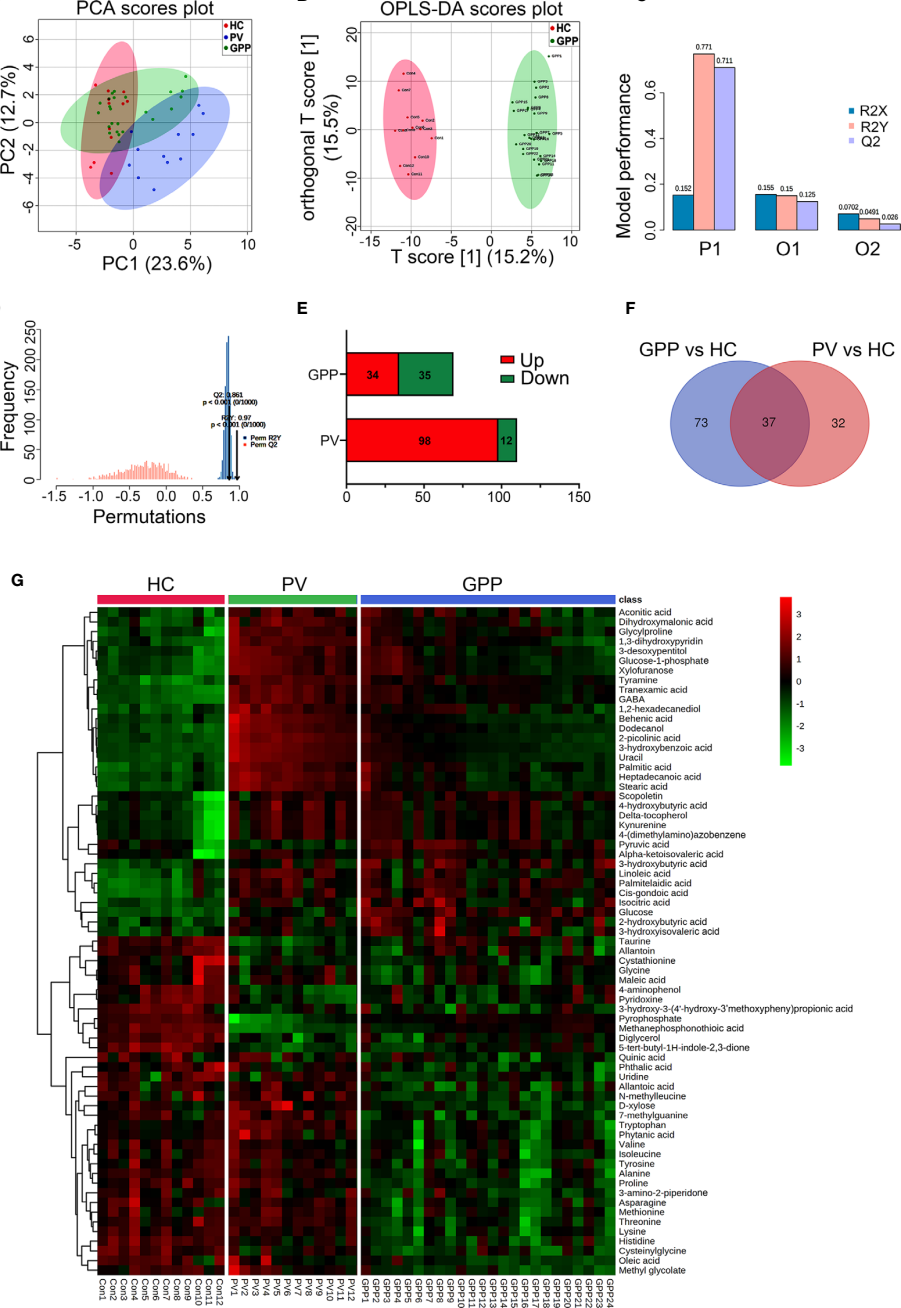
Non-targeted metabolomics profiling analysis for serum from GPP, PV and HC. **(A)** PCA scores plot of non-targeted GC-MS from the GPP (n = 24), PV (n = 12) and HC (n = 12) groups. **(B)** OPLS-DA score plots from the GPP (n =24) and HC (n = 12) groups. **(C)** Parameters of the OPLS-DA model (R^2^Y = 0.771, Q^2^Y = 0.711). **(D)** 1000-fold cross-validation of the OPLS-DA model by label permutation. *P* < 0.001 for R^2^Y and Q^2^Y. **(E)** Number of significantly different metabolites between GPP *vs* HC and PV *vs* HC. **(F)** The shared differential metabolites in the GPP *vs* HC and PV *vs* HC were visualized by Venn diagram. **(G)** Heatmap representing the contents of 69 differential metabolites, showing clear separation of GPP from PV and HC.

363 metabolites were identified using GC-MS. The PCA scoring plot showed a distinct separation in samples of GPP *vs* HC, and PV *vs* HC (**Figure 2A**). **Figure 2B** also showed that the GPP patients and HC are well distinguished by the OPLS-DA scores plot. The OPLS-DA model (R^2^Xcum = 0.152, R^2^Ycum= 0.771, Q^2^cum= 0.711) generated by 10-fold cross-validation indicated a satisfied robustness of the model (**Figure 2C**). To further validate this OPLS-DA model, a 1000-time permutation test was performed. **Figure 2D** showed that the empirical *P*-values for R^2^Y and Q^2^ were all < 0.001. In addition, PV patients and HC were well separated by OPLS-DA model (**Supplementary Figure S1**). These results demonstrated that the serum metabolic perturbation can distinguish GPP and PV patients from HC.

Based on VIP-values (VIP > 1) in the OPLS-DA model and t-test (*P* < 0.05) separating GPP patients from HC, a set of 69 metabolites were screened as significantly changed in GPP patients compared to HC, including 34 increased metabolites and 35 decreased metabolites (**Table 2**). Compared with HC, 110 metabolites were detected in PV group, including 98 increased metabolites and 12 decreased metabolites (**Figure 2E**). Furthermore, there were 37 common metabolites in the comparisons between GPP *vs* HC group and PV *vs* HC group (**Figure 2F**). **Figure 2G** showed a heatmap visualizing the metabolic perturbations in GPP.

**Table 2:** Significantly altered serum metabolites in GPP patients.

Classification	Metabolite	VIP^*^	*P*-value^†^	FC^‡^	Trend	AUC
Amino acids, peptides, and analogues	4-aminobutyric acid (GABA)	2.29	5.97E-07	2.33	Up	1.000
	Glycyl proline	1.03	4.72E-04	1.27	Up	0.917
	Tranexamic acid	2.41	3.53E-07	2.48	Up	1.000
	Tyramine	1.52	2.40E-08	1.44	Up	1.000
	Kynurenine	1.63	9.41E-04	1.71	Up	0.910
	3-amino-2-piperidone	1.48	1.07E-02	0.56	Down	0.851
	Allantoic acid	1.40	8.77E-03	0.59	Down	0.806
	Glycine	1.44	6.61E-03	0.56	Down	0.840
	Alanine	1.43	4.11E-05	0.65	Down	0.944
	Asparagine	1.53	7.65E-03	0.50	Down	0.826
	Cystathionine	1.21	6.82E-03	0.64	Down	0.799
	Histidine	2.78	8.68E-06	0.29	Down	0.962
	Isoleucine	1.14	1.07E-02	0.66	Down	0.781
	Lysine	1.65	5.97E-05	0.56	Down	0.927
	Methionine	1.16	1.10E-02	0.65	Down	0.760
	Proline	1.67	2.04E-05	0.59	Down	0.951
	Threonine	1.68	4.68E-04	0.52	Down	0.931
	Tyrosine	1.33	3.96E-03	0.65	Down	0.823
	Valine	1.16	1.85E-03	0.71	Down	0.833
	Taurine	1.99	1.20E-03	0.43	Down	0.875
	Tryptophan	1.21	2.35E-02	0.68	Down	0.778
	Cysteine-glycine	2.37	2.04E-05	0.37	Down	0.944
	N-methylleucine	1.99	4.31E-02	0.24	Down	0.806
Benzene and substituted derivatives	4-aminophenol	1.39	4.68E-04	0.68	Down	0.889
	3-hydroxybenzoic acid	1.83	4.68E-04	1.92	Up	0.993
	Phthalic acid	1.61	2.29E-03	0.56	Down	0.875
Organic acids and derivatives	Pyruvic acid	1.96	3.29E-02	2.25	Up	0.760
	2-hydroxybutyric acid	1.53	4.51E-02	2.08	Up	0.844
	3-hydroxybutyric acid	2.30	2.65E-02	5.02	Up	0.861
	Maleic acid	1.31	1.88E-02	0.57	Down	0.799
	Alpha-ketoisovaleric acid	1.56	6.61E-03	1.58	Up	0.788
	Aconitic acid	1.21	7.65E-03	1.47	Up	0.844
	Isocitric acid	1.34	7.53E-03	1.69	Up	0.931
Organic oxygen compounds	Glucose	2.70	6.94E-06	3.32	Up	1.000
	Glucose-1-phosphate	1.60	5.11E-03	1.80	Up	0.927
	D-xylose	1.28	6.16E-03	0.66	Down	0.844
	Quinic acid	2.14	4.95E-02	0.17	Down	0.760
	Phytanic acid	1.23	7.65E-03	0.67	Down	0.802
Lipids and lipid-like molecules	3-hydroxyisovaleric acid	1.47	4.85E-02	2.03	Up	0.837
	4-hydroxybutyric acid	1.25	7.95E-04	1.41	Up	0.868
	Behenic acid	1.03	3.57E-02	1.34	Up	0.809
	Cis-gondoic acid	2.18	9.71E-04	2.42	Up	0.927
	Heptadecanoic acid	1.03	1.49E-02	1.34	Up	0.868
	Palmitelaidic acid	2.66	1.69E-03	3.49	Up	0.899
	Palmitic acid	1.22	1.35E-03	1.38	Up	0.941
	Stearic acid	1.00	6.61E-03	1.28	Up	0.913
	Oleic acid	2.18	4.32E-05	0.37	Down	0.858
	Delta-tocopherol	1.54	9.71E-04	1.63	Up	0.885
	Linoleic acid	1.44	4.05E-03	1.60	Up	0.882
	Dodecanol	1.80	6.86E-05	1.78	Up	0.972
Hydroxycoumarins	Scopoletin	1.86	1.40E-04	1.88	Up	0.931
Non-metal pyrophosphates	Pyrophosphate	2.24	3.82E-07	0.43	Down	0.983
Organoheterocyclic compounds	Allantoin	1.74	6.61E-03	0.52	Down	0.830
	7-methylguanine	1.63	9.41E-04	0.52	Down	0.854
	2-picolinic acid	1.21	3.96E-03	1.41	Up	0.931
	Pyridoxine	1.21	1.13E-03	0.73	Down	0.851
	Uracil	1.72	2.43E-03	1.89	Up	0.969
Unclassified	4-(dimethylamino)azobenzene	1.63	1.40E-03	1.73	Up	0.892
	Uridine	1.33	4.68E-04	0.63	Down	0.865
	1,3-dihydroxypyridine	1.05	3.83E-03	1.31	Up	0.854
	3-desoxypentitol	1.37	1.61E-02	1.68	Up	0.861
	Dihydroxymalonic acid	1.04	6.82E-03	1.29	Up	0.844
	1,2-Hexadecanediol	1.30	6.60E-04	1.48	Up	0.896
	Xylofuranose	1.63	2.94E-03	1.81	Up	0.948
	3-hydroxy-3-(4’-hydroxy-3’-methoxyphenyl)propionic acid	1.32	6.58E-04	0.69	Down	0.882
	5-tert-butyl-1H-indole-2,3-dione	1.60	1.25E-06	0.65	Down	0.962
	Diglycerol	1.12	2.04E-05	0.78	Down	0.972
	Methanephosphonothioic acid	1.59	5.24E-08	0.67	Down	0.979
	Methyl glycolate	1.35	2.04E-05	0.70	Down	0.944

^*^Variable importance in the projection (VIP) was acquired from OPLS-DA with a threshold of 1.0.

^†^P-values were corrected by Benjamini-Hochberg method with FDR < 5%.

^‡^FC (Fold change) was obtained from the arithmetic mean values of each group. FC > 1 suggests a relatively higher concentration present in model cell while FC < 1 represents a lower concentration compared with the controls.

### Amino Acid Starvation Is Characteristic of GPP Circulation

**Figure 3:**
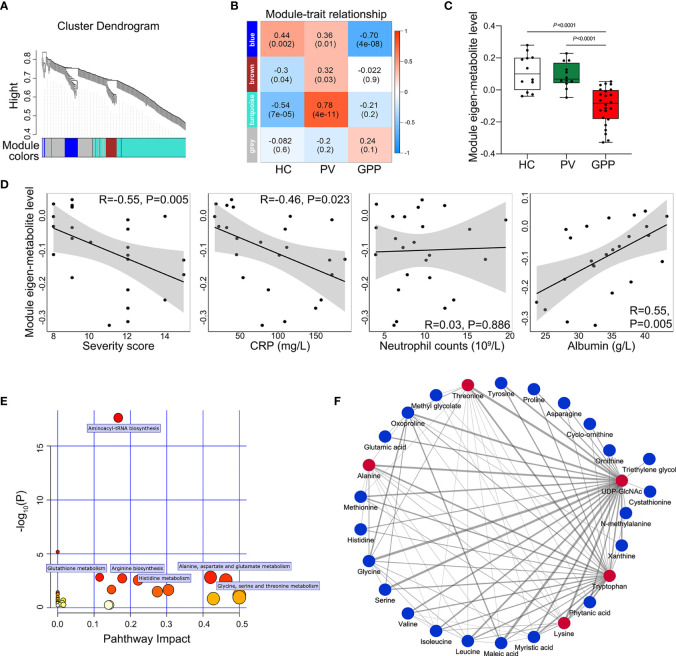
Metabolite co-abundant network analysis. **(A)** Network cluster dendrogram constructed by hierarchical clustering of the metabolomic dataset. Three network modules were demonstrated in different colors. **(B)** Heatmap of the correlation between module eigen-metabolites and disease status. The heatmap cell color signified the correlation coefficients (red = positive, blue = negative). In the cells, the figure without parentheses showed the correlation coefficients. The *P*-values were indicated below in parentheses. **(C)** The eigen-metabolite values of blue module separate GPP (n = 24) from both HC (n = 12) and PV (n =12) groups by one way ANOVA. **(D)** The correlation between blue module eigen-metabolite values and GPP clinical parameters including severity scores, GPP levels, neutrophil counts, and albumin levels, as assessed by Pearson’s correlation. **(E)** Summary of pathway topology analysis of the metabolites within the blue module, with the target pathways being selected and labeled. **(F)** Network of metabolites within the blue module was constructed by Cytoscape (edge weight > 0.3. The metabolites and connections between them are represented by nodes and edges. The red color indicates the top 5 metabolites with the highest number of connections. Strong connections are visualized as wider lines.

To further identify clusters of metabolites that might be associated with GPP, a metabolite co-abundance network was constructed on the 363-metabolite data set using WGCNA method. Using average linkage hierarchical clustering, a total of 3 modules of co-abundant metabolites, ranging from 31 to 195 metabolites, were constructed (**Figure 3A**). To determine disease-relevant modules associated with GPP, we investigated the module-trait relationships by computing the biweight midcorrelations between each module eigenmetabolite (the module representative which summarises metabolite profiles within the module) and disease status. The blue module, comprising 37 metabolites, was negatively correlated with GPP phenotype, but positively correlated with PV and HC group (**Figure 3B**). The module eigen-metabolite (ME) values were significantly decreased in GPP samples compared to PV and HC samples (**Figure 3C**). Within GPP group, ME values showed negative correlation with severity scores and CRP levels, and positive correlation with albumin levels, but no correlation with neutrophil counts (**Figure 3D**).

To reveal potential biological functions of metabolite within the blue module, pathway enrichment analysis was conducted *via* an interactive visualization framework. Pathway enrichment and topology analysis was performed to select the potential target pathways. As shown in **Figure 3E**, amino acid metabolism constituted all the selected 6 biological pathways.

The connections between the metabolites in the blue module were selected from the metabolite network with an edge weight > 0.3, generating a network of 27 nodes and 88 edges. Cytoscape was used to visualize the networks highlighting edge weight and degree (**Figure 3F**). Top 5 metabolites (UDP-GlcNAc, tryptophan, threonine, alanine and lysine) demonstrated the highest number of connections, highlighting them as potentially co-regulated hub metabolites in GPP.

**Figure 4:**
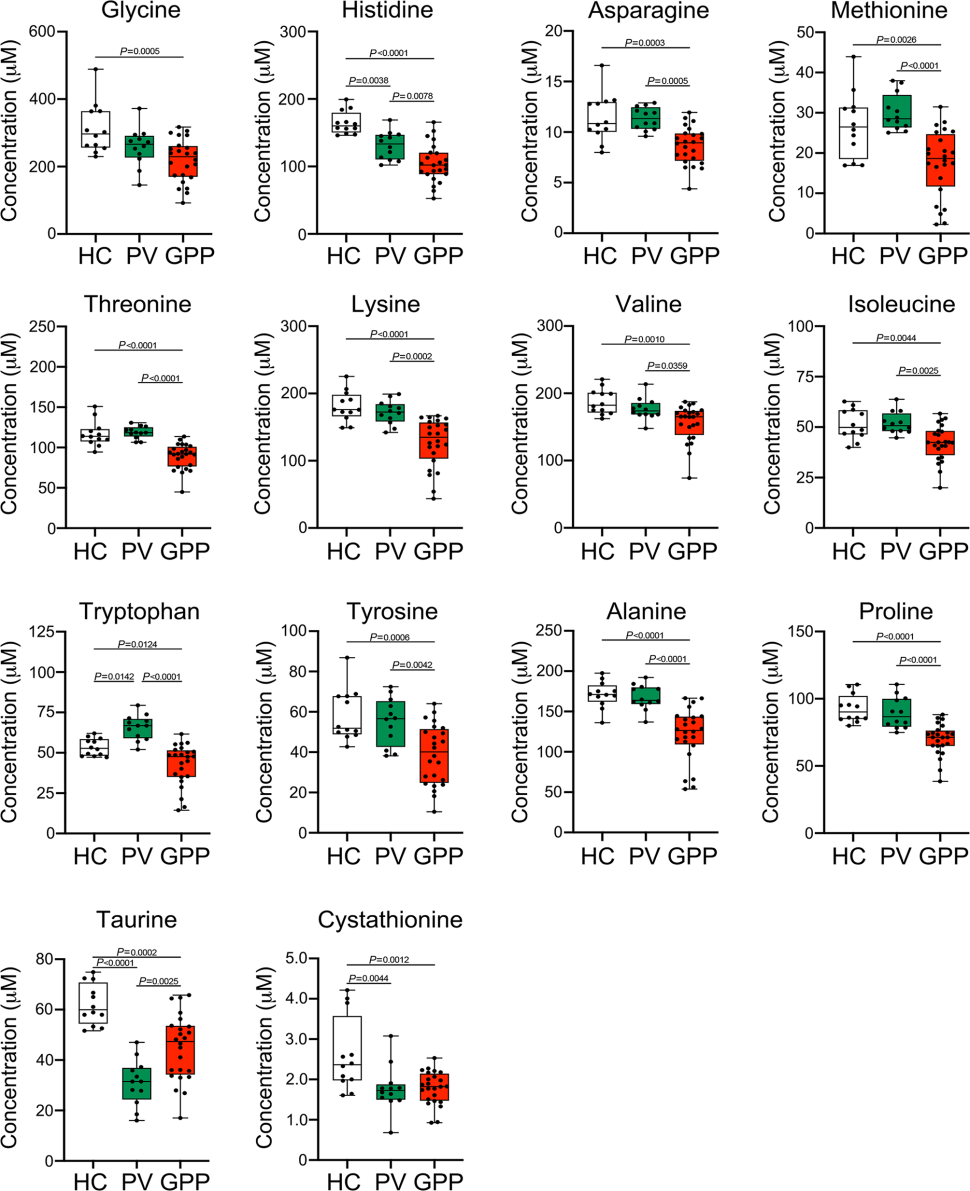
Amino acid alterations in GPP serum identified by targeted metabolomics. Boxplot of the 14 differential amino acids including glycine, histidine, asparagine, methionine, threonine, lysine, valine, isoleucine, tryptophan, tyrosine, alanine, proline, taurine and cystathionine in GPP (n = 24), PV (n = 12) and HC (n =12) serum. Data are presented as mean ± SD, and are assessed by One-way ANOVA and Dunnett’s *post hoc* test.

Our non-targeted metabolomic analysis showed significant perturbations of amino acids in sera of GPP patients, suggesting a possible role of amino acid metabolism in GPP. Next, the levels of amino acids in GPP patients, PV patients and HC were confirmed by amino acid-targeted metabolomics. The levels of glycine, histidine, asparagine, methionine, threonine, lysine, valine, isoleucine, tryptophan, tyrosine, alanine, proline, taurine and cystathionine were significantly decreased in GPP patients compared to HC, while only histidine, tryptophan, taurine and cysathionine were significantly different between PV and HC (**Figure 4**).

**Figure 5:**
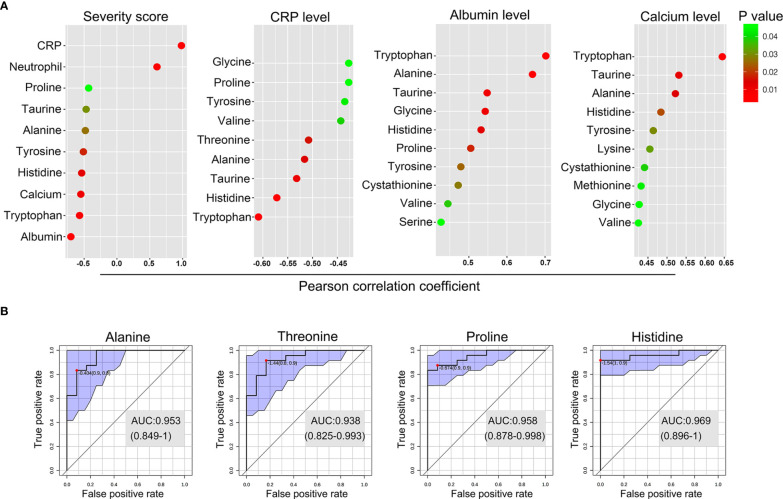
Biomarker analysis of the amino acid in GPP. **(A)** Pearson partial correlation analysis (adjusted for gender, age and BMI) between 14 amino acids and clinical traits of GPP including JDA severity scores, CRP levels, albumin levels and calcium levels. **(B)** The performance prediction of metabolites as potential biomarkers is demonstrated as ROC curves. Four metabolites of AUC values higher than 0.9 were shown.

We next investigated the correlation between the levels of the perturbed amino acids and clinical and serological markers of disease activity in GPP using Pearson partial correlation analysis (adjusted for gender, age and BMI). Tryptophan, histidine, tyrosine, alanine and taurine were significantly correlated with all the GPP clinical parameters (**Figure 5A**). In particular, the most strongly correlated amino acid was tryptophan, suggesting an inflammation-associated tryptophan catabolism.

Next, multivariate receiver operating characteristic (ROC) curve analysis was performed to separate GPP from HC. A high-efficiency prediction of potential biomarkers was well achieved, with the area under the curve (AUC) values of 4 amino acids (alanine, threonine, proline and histidine) ranging from 0.90 to 1.00 (**Figure 5B**).

### Amino Acid Starvation Dampens SAA-Triggered IL-1β Production in Monocytes

**Figure 6:**
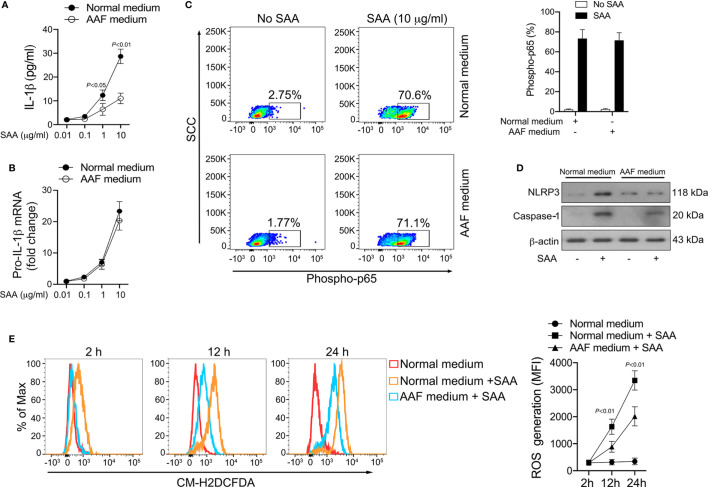
Inhibition of SAA-induced IL-1β by amino acid starvation. **(A, B)** Healthy monocytes were exposed to normal complete medium or AAF medium in the presence of SAA for 24 h IL-1β secretion was examined by ELISA (n = 6), while pro-IL-1β mRNA was measured by real-time RT-PCR (n = 6). **(C)** Healthy monocytes were exposed to normal complete medium or AAF medium for 12 h, and then were stimulated with SAA (10 μg/ml) for 15 min. The phosphorylation of NF-κB p65 was measured using flow cytometry. Representative dot plots and mean phospho-p65^+^ cells (%); mean ± SD were acquired from four independent analyses. **(D)** Healthy monocytes were cultured with 10 μg/ml SAA for 24 h plus normal complete medium or AAF medium. NLRP3 and active caspase-1 were examined using Western blot. Representative figures of four independent analyses are shown. **(E)** Healthy monocytes were cultured with SAA for 2 h, 12 h and 24 h in the presence of normal complete medium or AAF medium. Then the melanocytes were stained by CM-H2DCFDA, and ROS generation was tested using flow cytometry. Representative histograms and mean fluorescence intensities (MFI) were acquired from four independent analyses.

Our results revealed that most of the amino acid species were significantly decreased in GPP serum, however, the role of the alteration in amino acid levels in GPP pathogenesis is unknown. It was therefore of interest to investigate whether amino acid limitation could influence SAA-induced IL-1β secretion from monocytes. To this aim, normal human monocytes were cultured in the presence of SAA in either amino acid-free (AAF) medium or normal complete medium. AAF medium incubation significantly dampened the SAA-induced IL-1β secretion (**Figure 6A**).

The production of mature IL-1β is mediated transcriptionally and post-transcriptionally. AAF medium incubation did not modify SAA-induced pro-IL-1β mRNA expression (**Figure 6B**). Consistently, SAA-triggered NF-κB activation was not influenced by AAF medium culturing (**Figure 6C**). These data suggested that amino acid starvation might reduce IL-1β secretion in monocytes on post-transcriptional level instead of transcriptional level. At the post-transcriptional level, AAF medium culturing modulated the expression of NLRP3 and active caspase-1 induced by SAA (**Figure 6D**), suggesting the activation of NLRP3 inflammasome by SAA could be suppressed by amino acid deficiency.

We previously reported that ROS was involved in SAA-mediated inflammasome activation and IL-1β release at the post-transcriptional level (15). We therefore examined the effect of amino acid starvation on SAA-induced ROS generation. As shown in **Figure 6E**, ROS formation in monocytes increased significantly following SAA stimulation, while AAF medium incubation partially abrogated this effect, suggesting that amino acid starvation might inhibit IL-1β production through ROS suppression.

### The Modulatory Effect of Amino Acid Starvation on IL-1β Release Is Dependent on GCN2

**Figure 7:**
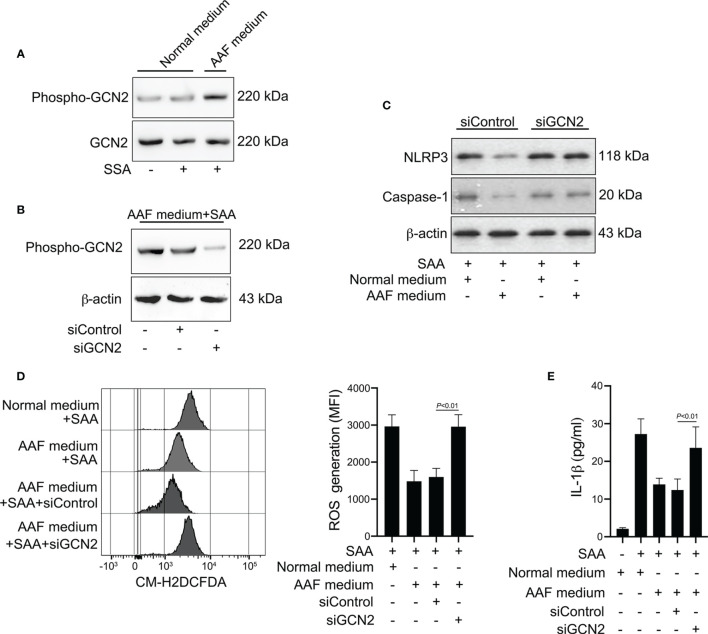
Involvement of GCN2 in the effects of amino acid starvation. **(A)** Healthy monocytes were stimulated with SAA in the presence of normal complete medium or AAF medium. Protein expression of phosphor-GCN2 and GCN2 were measured by Western blot. Data are from one experiment representative of four. **(B)** Healthy monocytes were transfected by siRNA targeting GCN2 (siGCN2) or a non-specific siRNA (siControl). GCN2 protein was examined by Western blot. Data are from one analysis representative of four. **(C)** siRNA-transfected monocytes were treated with 10 μg/ml SAA for 24 h in the presence of normal complete medium or AAF medium. NLRP3 and the active caspase-1 were measured by Western blot. Representative blots of four independent analyses are shown. **(D)** siRNA-transfected monocytes were then cultured with 10 μg/ml SAA under AAF condition for 24 h Then the melanocytes were stained by CM-H2DCFDA, and ROS generation was tested using flow cytometry. Representative histograms and MFI were acquired from four independent analyses. **(E)** siRNA-transfected monocytes were then stimulated by SAA (10 μg/ml) plus AAF medium, and culture supernatants were tested for IL-1β. The data represent the mean ± SD of four analyses.

The sensing of amino acid deficiency by GCN2 and triggering of downstream events is referred to as amino acid response (AAR) pathway (31, 32). Given that amino acid starvation decreased the ROS generation and IL-1β production induced by SAA, we tested whether amino acid depletion could activate the GCN2 in monocytes. While phosphorylation of GCN2 was not increased by SAA stimulation, co-stimulation with SAA and AAF medium induced GCN2 activation in monocytes (**Figure 7A**). To examine the role of GCN2 in the effect of amino acid starvation on NLRP3 inflammasome and IL-1β production, we inhibited GCN2 expression by siRNA (**Figure 7B**). Knockdown of GCN2 rescued the reduction in NLRP3 and caspase-1 expression induced by AAF medium (**Figure 7C**). Inhibition of GCN2 expression restored the ROS levels decreased by AAF medium (**Figure 7D**). Consistently, the reduction of IL-1β by AAF medium was also rescued by GCN2 knockdown (**Figure 7E**). These data indicated that the suppressed NLRP3 inflammasome, ROS generation and IL-1β production by amino acid starvation is dependent on GCN2.

## Discussion

Metabolomics focuses on the relationship between disease and metabolic perturbation, based on techniques such as UPLC-MS/MS and GC-MS (17). In the present study, our results identified perturbations in circulating metabolome of GPP patients compared with healthy controls using non-targeted GC-MS and targeted UPLC-MS/MS. In particular, we noted differences in metabolites related to amino acid metabolism. Although a clear linkage between PV and perturbed circulating amino acids has been established in several studies (18–22), there is a paucity of information on GPP. A significantly upregulation of most amino acids in PV has been reported, paralleling with the disease severity score (18, 21). Moreover, treatment of psoriasis shifted the disease-specific trends in plasma metabolites toward that of normal subjects. In contrast, a downregulation of glucogenic amino acids, branched-chain amino acids, sulfur-containing amino acids and essential amino acids in GPP were observed in the current study. The sharp contrast in metabolic shift in PV and GPP suggested that GPP might not be, from a metabolomic view, an escalation of PV along a common metabolic spectrum, but a particular variant of psoriasis.

The responsible mechanisms for the observed shifts in circulating metabolites are largely unknown. It was speculated that increased levels of amino acids in PV is due to enhanced protein synthesis during keratinocyte hyperproliferation (18). The acute onset of GPP is frequently accompanied by elevated levels of acute-phase protein and decreased levels of serum albumin. The catabolic rates of patients during acute-phase reaction are faster than the anabolic rates (33). This phenomenon might be associated with inflammatory cytokines like IL-1β, IL-6 and TNF-α (34). We thus speculated that GPP is related to elevated metabolic level in the liver. Triggered by systemic inflammatory cytokines, amino acids are transferred to the liver for protein synthesis, like acute-phase proteins, leading to downregulation of serum amino acid levels in GPP.

Granulocyte and monocyte apheresis (GMA) has been recently used in GPP treatment. During GMA treatment, circulating granulocytes and monocytes are eliminated from bloodstream (35). GMA has shown clinical efficacy in patients with GPP (36, 37). In addition, a significant downregulation in IL-1β, IL-6, and TNF-α has been observed following GMA therapy (38). Monocytes take part in a variety of immune function processes including cytokine production, phagocytosis and antigen presentation. Circulating monocytes also migrate into peripheral tissues to differentiate into macrophages or dendritic cells. The dramatic immunomodulatory effects of GMA on GPP supports the speculation that blood monocytes, as a major source of inflammatory cytokines, might be an essential part of innate immune response of GPP.

A principal determinant of GPP appears to be a cytokine storm corresponding to that seen in other sterile and non-sterile inflammation. Clinical markers of cytokine storm include increased circulating CRP and erythrocyte sedimentation rate. Observational studies have noted upregulation of a variety of circulating cytokines in GPP (39, 40). It has been shown that GPP lesions furnished higher IL-1β and IL-1RN mRNA expression than PV (2). Recently, it was reported that patients with severe pustular psoriasis showed substantial clinical response to anti-IL-1β gevokizumab and canakinumab (6, 7), suggesting a critical involvement of IL-1β in GPP pathogenesis. Despite IL-1β mRNA is elevated in GPP skin, it is not conclusive whether IL-1β protein expression is also upregulated in circulating myeloid cells like monocytes. Here, we showed that blood monocytes from GPP patients secret increased pro-inflammatory IL-1β, which parallels with the speculation that circulating monocytes boost a cytokine storm during GPP progression.

One critical question is how IL-1β secretion is triggered and regulated in GPP monocytes. Our previous studies have shown that SAA stimulated NLRP3 inflammasome pathway in keratinocytes resulting into IL-1β upregulation (15). Here, our data suggested that monocyte was another target of SAA. In systemic inflammation, the synthetic capacity of the liver has been focused on the production of SAA and other acute-phase proteins (41). Moreover, previous studies demonstrated that SAA was increased in psoriatic epidermis (42, 43). Here, we speculated that both liver and inflamed skin might be source of increased SAA in GPP patients. Considering that IL-1β is a trigger for SAA production, SAA might act as a connection in monocyte function and acute-phase reaction, forming a feed-forward and vicious loop in GPP.

Amino acid are not only vital nutrients for cellular homeostasis but also as the signaling molecules. To maintain cellular homeostasis, the availability of amino acids should be efficiently sensed (44). The amino acid starvation environment is sensed by GCN2, which is referred to as AAR pathway. During amino acid scarcity, uncharged tRNAs accumulate in the cell and activate GCN2 to launch a downstream pathway (32). In our study, the WGCNA and pathway analysis revealed aminoacyl-tRNA biosynthesis as the metabolic pathway most strongly associated with GPP, suggesting the activation of AAR pathway by amino acid decrease.

The NLRP3 inflammasome activation is dependent on both NF-κB pathway and generation of intracellular ROS (10). Previously, it has been shown that GCN2 knockout mice produced more ROS (31). Consistently, in the present study, we demonstrated that amino acid starvation could dampen the ROS generation. ROS-associated signaling pathway might be involved in the pathogenesis of systemic inflammation like psoriasis (45). Therefore, we hypothesize that amino acid abundance might be a promising therapeutic target in ROS-associated pathogenesis in a vast array of inflammatory disorders.

**Figure 8:**
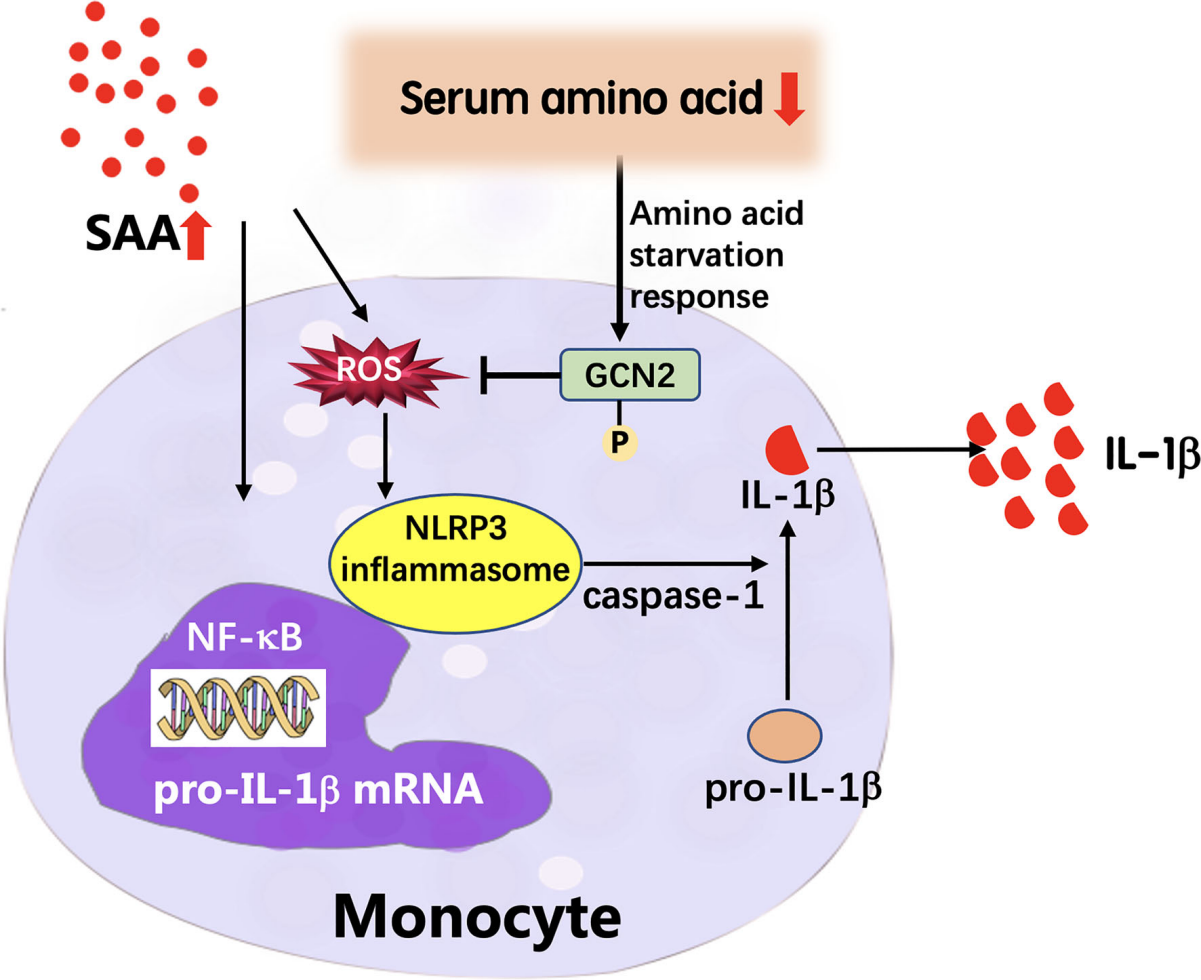
Proposed model of the feedback mechanism by which low amino acids suppress IL-1β expression in GPP. In GPP, IL-1β and other inflammatory cytokines induce dramatic upregulation of acute-phase proteins including SAA. SAA activates the NLRP3 inflammasome pathway and secretion of mature IL-1β from blood monocytes as an amplification loop; on the other hand, the acute-phase reaction results into amino acid starvation in circulation. Low amino acids induce activation of GCN2, which decreases ROS levels, thereby inhibiting NLRP3 inflammasome pathway and IL-1β release, as a negative feedback mechanism.

In summary, we explored the GPP serum metabolic profile using a global metabolomics approach. The levels of circulating amino acids were decreased in GPP and were useful in monitoring the severity of the disease. In functional study, we demonstrated that amino acid deficiency suppressed the IL-1β production from monocytes through GCN2-AAR pathway. Our data revealed a novel regulatory mechanism of circulating metabolomic signature on innate immune responses in GPP (**Figure 8**). These data might contribute to the comprehension of immunopathogenesis of GPP, and help to design therapeutic intervention against this severe inflammatory disease.

## Data Availability Statement

The raw data supporting the conclusions of this article will be made available by the authors, without undue reservation.

## Ethics Statement

The studies involving human participants were reviewed and approved by The Ethics Committee of Shanghai Skin Disease Hospital of Tongji University. The patients/participants provided their written informed consent to participate in this study.

## Author Contributions

NY, CP, WC, and ZS performed experiments. NY analyzed data. JZ was involved in sample and reagent acquisition. NY was involved in the conceptual development of the study and wrote the manuscript. YD, SZ, and YS coordinated and supervised the entire study. All authors contributed to the article and approved the submitted version.

## Funding

This work was funded from National Natural Science Foundation of China (No. 81972934, 81872522, 82073429), Innovation Program of Shanghai Municipal Education Commission (No. 2019-01-07-00-07-E00046), the Program of Science and Technology Commission of Shanghai Municipality (No. 18140901800), Excellent Subject Leader Program of Shanghai Municipal Commission of Health and Family Planning (No. 2018BR30), Clinical Research Plan of SHDC (No. SHDC2020CR1014B, SHDC12018X06) and Program of Shanghai Academic Research Leader (No. 20XD1403300).

## Conflict of Interest

The authors declare that the research was conducted in the absence of any commercial or financial relationships that could be construed as a potential conflict of interest.

## Publisher’s Note

All claims expressed in this article are solely those of the authors and do not necessarily represent those of their affiliated organizations, or those of the publisher, the editors and the reviewers. Any product that may be evaluated in this article, or claim that may be made by its manufacturer, is not guaranteed or endorsed by the publisher.

## Supplementary Material

Supplementary Figure S1 **(A)** OPLS-DA score plots from the PV (n =12) and HC (n = 12) groups. **(B)** Parameters of the OPLS-DA model (R^2^Y = 0.901, Q^2^Y = 0.877). **(C)** 1000-fold cross-validation of the OPLS-DA model by label permutation. *P* < 0.001 for R^2^Y and Q^2^Y.

## References

[1] Hoegler KM John AM Handler MZ Schwartz RA. Generalized Pustular Psoriasis: A Review and Update on Treatment. J Eur Acad Dermatol Venereol (2018) 32:1645–51. 10.1111/jdv.14949 29573491

[2] Johnston A Xing X Wolterink L Barnes DH Yin Z Reingold L. IL-1 and IL-36 are Dominant Cytokines in Generalized Pustular Psoriasis. J Allergy Clin Immunol (2017) 140:109–20. 10.1016/j.jaci.2016.08.056 PMC5494022 28043870

[3] Boehner A Navarini AA Eyerich K. Generalized Pustular Psoriasis - a Model Disease for Specific Targeted Immunotherapy, Systematic Review. Exp Dermatol (2018) 27:1067–77. 10.1111/exd.13699 29852521

[4] Lowes MA Suarez-Farinas M Krueger JG. Immunology of Psoriasis. Annu Rev Immunol (2014) 32:227–55. 10.1146/annurev-immunol-032713-120225 PMC4229247 24655295

[5] Cai Y Xue F Quan C Qu M Liu N Zhang Y. A Critical Role of the IL-1beta-IL-1r Signaling Pathway in Skin Inflammation and Psoriasis Pathogenesis. J Invest Dermatol (2019) 139:146–56. 10.1016/j.jid.2018.07.025 PMC6392027 30120937

[6] Skendros P Papagoras C Lefaki I Giatromanolaki A Kotsianidis I Speletas M. Successful Response in a Case of Severe Pustular Psoriasis After Interleukin-1beta Inhibition. Br J Dermatol (2017) 176:212–5. 10.1111/bjd.14685 27105586

[7] Mansouri B Richards L Menter A. Treatment of Two Patients With Generalized Pustular Psoriasis With the Interleukin-1beta Inhibitor Gevokizumab. Br J Dermatol (2015) 173:239–41. 10.1111/bjd.13614 25495649

[8] Hume DA Irvine KM Pridans C. The Mononuclear Phagocyte System: The Relationship Between Monocytes and Macrophages. Trends Immunol (2019) 40:98–112. 10.1016/j.it.2018.11.007 30579704

[9] Hsi ED Remick DG. Monocytes are the Major Producers of Interleukin-1 Beta in an Ex Vivo Model of Local Cytokine Production. J Interferon Cytokine Res (1995) 15:89–94. 10.1089/jir.1995.15.89 7648438

[10] Zheng D Liwinski T Elinav E. Inflammasome Activation and Regulation: Toward a Better Understanding of Complex Mechanisms. Cell Discov (2020) 6:36. 10.1038/s41421-020-0167-x PMC7280307 32550001

[11] Wang L Sharif H Vora SM Zheng Y Wu H. Structures and Functions of the Inflammasome Engine. J Allergy Clin Immunol (2021) 147:2021–9. 10.1016/j.jaci.2021.04.018 PMC8597577 34092352

[12] Sodin-Semrl S Zigon P Cucnik S Kveder T Blinc A Tomsic M. Serum Amyloid A in Autoimmune Thrombosis. Autoimmun Rev (2006) 6:21–7. 10.1016/j.autrev.2006.03.006 17110312

[13] Migita K Izumi Y Jiuchi Y Kozuru H Kawahara C Izumi M. Effects of Janus Kinase Inhibitor Tofacitinib on Circulating Serum Amyloid A and Interleukin-6 During Treatment for Rheumatoid Arthritis. Clin Exp Immunol (2014) 175:208–14. 10.1111/cei.12234 PMC3892412 24665995

[14] Dogan S Atakan N. Is Serum Amyloid A Protein a Better Indicator of Inflammation in Severe Psoriasis? Br J Dermatol (2010) 163:895–6. 10.1111/j.1365-2133.2010.09907.x 20553266

[15] Yu N Liu S Yi X Zhang S Ding Y. Serum Amyloid A Induces Interleukin-1beta Secretion From Keratinocytes *via* the NACHT, LRR and PYD Domains-Containing Protein 3 Inflammasome. Clin Exp Immunol (2015) 179:344–53. 10.1111/cei.12458 PMC4298410 25231464

[16] Kang J Zhu L Lu J Zhang X. Application of Metabolomics in Autoimmune Diseases: Insight Into Biomarkers and Pathology. J Neuroimmunol (2015) 279:25–32. 10.1016/j.jneuroim.2015.01.001 25669996

[17] Yan J. Identifying Biomarkers in Human Psoriasis: Revealed by a Systems Metabolomics Approach. Br J Dermatol (2017) 176:555–7. 10.1111/bjd.15249 PMC5356491 28300304

[18] Kamleh MA Snowden SG Grapov D Blackburn GJ Watson DG Xu N. LC-MS Metabolomics of Psoriasis Patients Reveals Disease Severity-Dependent Increases in Circulating Amino Acids That are Ameliorated by Anti-TNFalpha Treatment. J Proteome Res (2015) 14:557–66. 10.1021/pr500782g PMC4286171 25361234

[19] Armstrong AW Wu J Johnson MA Grapov D Azizi B Dhillon J. Metabolomics in Psoriatic Disease: Pilot Study Reveals Metabolite Differences in Psoriasis and Psoriatic Arthritis. F1000Res (2014) 3:248. 10.12688/f1000research.4709.1 25580230 PMC4288418

[20] Kang H Li X Zhou Q Quan C Xue F Zheng J. Exploration of Candidate Biomarkers for Human Psoriasis Based on Gas Chromatography-Mass Spectrometry Serum Metabolomics. Br J Dermatol (2017) 176:713–22. 10.1111/bjd.15008 27564527

[21] Ottas A Fishman D Okas TL Kingo K Soomets U. The Metabolic Analysis of Psoriasis Identifies the Associated Metabolites While Providing Computational Models for the Monitoring of the Disease. Arch Dermatol Res (2017) 309:519–28. 10.1007/s00403-017-1760-1 PMC5577063 28695330

[22] Li SS Liu Y Li H Wang LP Xue LF Yin GS. Identification of Psoriasis Vulgaris Biomarkers in Human Plasma by non-Targeted Metabolomics Based on UPLC-Q-TOF/Ms. Eur Rev Med Pharmacol Sci (2019) 23:3940–50. 10.26355/eurrev_201905_17823 31115022

[23] Sorokin AV Domenichiello AF Dey AK Yuan ZX Goyal A Rose SM. Bioactive Lipid Mediator Profiles in Human Psoriasis Skin and Blood. J Invest Dermatol (2018) 138:1518–28. 10.1016/j.jid.2018.02.003 PMC6121727 29454560

[24] Chen C Hou G Zeng C Ren Y Chen X Peng C. Metabolomic Profiling Reveals Amino Acid and Carnitine Alterations as Metabolic Signatures in Psoriasis. Theranostics (2021) 11:754–67. 10.7150/thno.51154 PMC7738860 33391503

[25] Li X Cheng J Shen Y Chen J Wang T Wen F. Metabolomic Analysis of Lung Cancer Patients With Chronic Obstructive Pulmonary Disease Using Gas Chromatography-Mass Spectrometry. J Pharm BioMed Anal (2020) 190:113524. 10.1016/j.jpba.2020.113524 32795777

[26] Langfelder P Horvath S. WGCNA: An R Package for Weighted Correlation Network Analysis. BMC Bioinf (2008) 9:559. 10.1186/1471-2105-9-559 PMC2631488 19114008

[27] Chong J Wishart DS Xia J. Using MetaboAnalyst 4.0 for Comprehensive and Integrative Metabolomics Data Analysis. Curr Protoc Bioinf (2019) 68:e86. 10.1002/cpbi.86 31756036

[28] Yang XH Zhang XX Jing Y Ding L Fu Y Wang S. Amino Acids Signatures of Distance-Related Surgical Margins of Oral Squamous Cell Carcinoma. EBioMedicine (2019) 48:81–91. 10.1016/j.ebiom.2019.10.005 31631041 PMC6838421

[29] Shalova IN Lim JY Chittezhath M Zinkernagel AS Beasley F Hernández-Jiménez E. Human Monocytes Undergo Functional Re-Programming During Sepsis Mediated by Hypoxia-Inducible Factor-1alpha. Immunity (2015) 42:484–98. 10.1016/j.immuni.2015.02.001 25746953

[30] Yamada T Wada A Itoh K Igari J. Serum Amyloid A Secretion From Monocytic Leukaemia Cell Line THP-1 and Cultured Human Peripheral Monocytes. Scand J Immunol (2000) 52:7–12. 10.1046/j.1365-3083.2000.00734.x 10886778

[31] Ravindran R Loebbermann J Nakaya HI Khan N Ma H Gama L. The Amino Acid Sensor GCN2 Controls Gut Inflammation by Inhibiting Inflammasome Activation. Nature (2016) 531:523–7. 10.1038/nature17186 PMC4854628 26982722

[32] Battu S Minhas G Mishra A Khan N. Amino Acid Sensing via General Control Nonderepressible-2 Kinase and Immunological Programming. Front Immunol (2017) 8:1719. 10.3389/fimmu.2017.01719 29321774 PMC5732134

[33] Su L Li H Xie A Liu D Rao W Lan L. Dynamic Changes in Amino Acid Concentration Profiles in Patients With Sepsis. PloS One (2015) 10:e0121933. 10.1371/journal.pone.0121933 25849571 PMC4388841

[34] Chang HR Bistrian B. The Role of Cytokines in the Catabolic Consequences of Infection and Injury. JPEN J Parenter Enteral Nutr (1998) 22:156–66. 10.1177/0148607198022003156 9586794

[35] Kanekura T. Clinical and Immunological Effects of Adsorptive Myeloid Lineage Leukocyte Apheresis in Patients With Immune Disorders. J Dermatol (2018) 45:943–50. 10.1111/1346-8138.14471 29782055

[36] Ikeda S Takahashi H Suga Y Eto H Etoh T Okuma K. Therapeutic Depletion of Myeloid Lineage Leukocytes in Patients With Generalized Pustular Psoriasis Indicates a Major Role for Neutrophils in the Immunopathogenesis of Psoriasis. J Am Acad Dermatol (2013) 68:609–17. 10.1016/j.jaad.2012.09.037 23332516

[37] Mizutani Y Fujii K Kawamura M Inoue M Mizutani YH Matsuyama K. Intensive Granulocyte and Monocyte Adsorption Apheresis for Generalized Pustular Psoriasis. J Dermatol (2020) 47:1326–9. 10.1111/1346-8138.15569 32860246

[38] Saniabadi AR Hanai H Takeuchi K Umemura K Nakashima M Adachi T. Adacolumn, an Adsorptive Carrier Based Granulocyte and Monocyte Apheresis Device for the Treatment of Inflammatory and Refractory Diseases Associated With Leukocytes. Ther Apher Dial (2003) 7:48–59. 10.1046/j.1526-0968.2003.00012.x 12921115

[39] Yamamoto M Imai Y Sakaguchi Y Haneda T Yamanishi K. Serum Cytokines Correlated With the Disease Severity of Generalized Pustular Psoriasis. Dis Markers (2013) 34:153–61. 10.1155/2013/702763 PMC3810235 23334651

[40] Nakamura S Hashimoto Y Igawa S Kajino M Nishi K Takahashi H. Childhood Generalized Pustular Psoriasis Treated by Preprandial Ciclosporin Administration: Serum Cytokine Pattern During the Course of the Disease. Clin Exp Dermatol (2009) 34:e1023–4. 10.1111/j.1365-2230.2009.03702.x 20055828

[41] Zhang Y Zhang J Sheng H Li H Wang R. Acute Phase Reactant Serum Amyloid A in Inflammation and Other Diseases. Adv Clin Chem (2019) 90:25–80. 10.1016/bs.acc.2019.01.002 31122611

[42] Yu N Zhang S Lu J Li Y Yi X Tang L. Serum amyloid A, An Acute Phase Protein, Stimulates Proliferative and Proinflammatory Responses of Keratinocytes. Cell Prolif (2017) 50:e12320. 10.1111/cpr.12320 PMC6529107 27910163

[43] Rooney P Connolly M Gao W McCormick J Biniecka M Sullivan O. Notch-1 Mediates Endothelial Cell Activation and Invasion in Psoriasis. Exp Dermatol (2014) 23:113–8. 10.1111/exd.12306 24330353

[44] Hotamisligil GS Erbay E. Nutrient Sensing and Inflammation in Metabolic Diseases. Nat Rev Immunol (2008) 8:923–34. 10.1038/nri2449 PMC2814543 19029988

[45] Gabr SA Al-Ghadir AH. Role of Cellular Oxidative Stress and Cytochrome C in the Pathogenesis of Psoriasis. Arch Dermatol Res (2012) 304:451–7. 10.1007/s00403-012-1230-8 22421888

