# Supplementary figures and images for: Circulating Metabolomic Signature in Generalized Pustular Psoriasis Blunts Monocyte Hyperinflammation by Triggering Amino Acid Response
Source: Front Immunol. 2021 Sep 8;12:739514. doi: 10.3389/fimmu.2021.739514 (PMC8455999; doi:10.3389/fimmu.2021.739514)

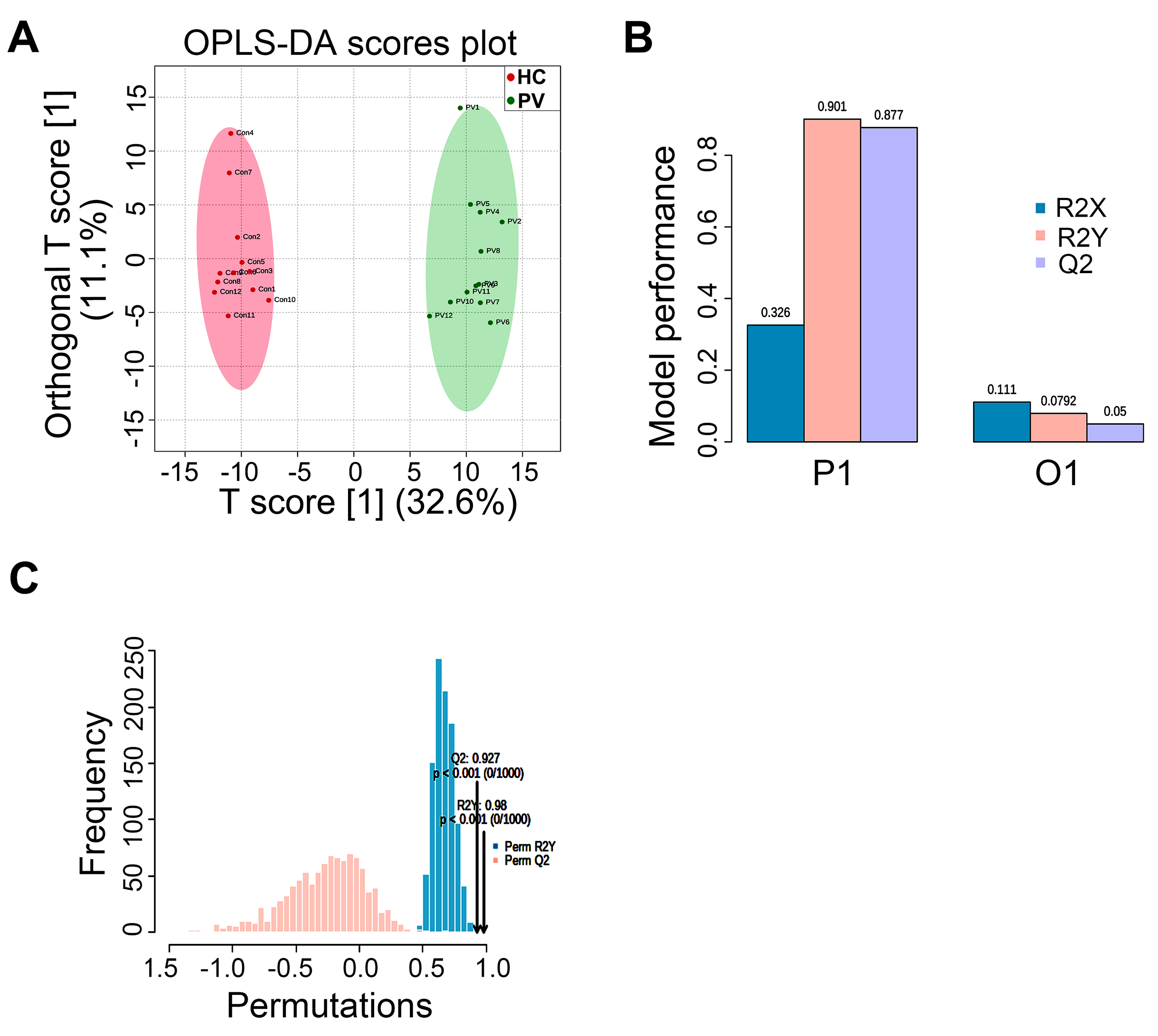

Supplement: Supplementary Figure S1 — (A) OPLS-DA score plots from the PV (n =12) and HC (n = 12) groups. (B) Parameters of the OPLS-DA model (R2Y = 0.901, Q2Y = 0.877). (C) 1000-fold cross-validation of the OPLS-DA model by label permutation. P < 0.001 for R2Y and Q2Y. [file Image_1.tif]
