# Supplementary material for: Circulating Metabolomic Signature in Generalized Pustular Psoriasis Blunts Monocyte Hyperinflammation by Triggering Amino Acid Response
Source: Front Immunol. 2021 Sep 8;12:739514. doi: 10.3389/fimmu.2021.739514 (PMC8455999; doi:10.3389/fimmu.2021.739514)
Supplement: Supplementary file 2 [file Table_1.docx]

**Table S1** Summary of pathway enrichment analysis of the metabolites within the brown module based on FDR *P*-values (< 0.05) and impact values (> 0.1).

|  | Total^*^ | Hits^†^ | –Log (*P)* | FDR *P*^‡^ | Impact^§^ |
| --- | --- | --- | --- | --- | --- |
| Glycine, serine and threonine metabolism | 33 | 6 | 4.1 | 2.01E-03 | 0.51 |
| Alanine, aspartate and glutamate metabolism | 28 | 5 | 3.5 | 4.75E-03 | 0.42 |
| Cysteine and methionine metabolism | 33 | 5 | 3.1 | 9.04E-03 | 0.40 |
| Arginine biosynthesis | 14 | 4 | 3.7 | 4.37E-03 | 0.18 |
| Aminoacyl-tRNA biosynthesis | 48 | 17 | 17.2 | 4.79E-16 | 0.17 |
| Glutathione metabolism | 28 | 5 | 3.5 | 4.75E-03 | 0.12 |

^*^Total is the amounts of compounds involved in the pathway.

^†^Hits is the actually matched number from the user uploaded data.

^‡^*P*-values were adjusted using Benjamini-Hochberg method with an FDR of less than 5%.

^§^Impact value is calculated from pathway topology analysis for comparison among diﬀerent pathways. It represents the cumulative percentage of importance for the matched metabolite nodes involved in a pathway. The importance of each metabolite node is calculated from centrality measures and represents the percentage with regard to the total pathway importance.
